# Network Pharmacology-Based Dissection of the Anti-diabetic Mechanism of *Lobelia chinensis*

**DOI:** 10.3389/fphar.2020.00347

**Published:** 2020-03-20

**Authors:** Qi Ge, Liang Chen, Yi Yuan, Lanlan Liu, Fan Feng, Peng Lv, Shangshang Ma, Keping Chen, Qin Yao

**Affiliations:** ^1^School of the Environment and Safety Engineering, Jiangsu University, Zhenjiang, China; ^2^Institute of Life Sciences, Jiangsu University, Zhenjiang, China; ^3^School of Food and Biological Engineering, Jiangsu University, Zhenjiang, China; ^4^School of Biological and Food Engineering, Suzhou University, Suzhou, China

**Keywords:** *Lobelia chinensis*, active ingredients, network pharmacology, anti-diabetic, anti-inflammatory

## Abstract

Diabetes mellitus (DM) is a chronic inflammatory disease, and the rapidly increasing DM is becoming a major problem of global public health. Traditional Chinese medicine (TCM) has a long history of treating diabetes. It has been developed and utilized because of its good efficacy and no toxic side effects. *Lobelia chinensis* is a traditional whole grass herbal. With the continuous deepening of pharmacological research on TCM, the active ingredients of *L. chinensis* are continuously revealed, which contained the alkaloids, flavonoids, flavonoid glycosides and amino acids that have the good effects of anti-inflammatory, anti-viral and anti-diabetic. In order to further explore the targets of active ingredients and its anti-diabetic mechanism, a feasible network pharmacology analysis model based on chemical, pharmacokinetic and pharmacological data was developed by network construction method to clarify the anti-diabetic mechanism of *L. chinensis*. The present study conducted by gas chromatography–mass spectrometer (GC/MS), which identified 208 metabolites of *L. chinensis*, of which 23 ingredients may have effective pharmacological effects after absorption, distribution, metabolism, and excretion (ADME) screening. Network pharmacological analysis on the active ingredients revealed that 5-hydroxymethylfurfural in *L. chinensis* affects the insulin resistance signaling pathway by acting on GSK3B, TNF, and MAPK1, acacetin affects the diabetic pathway by acting on INSR, DPP4, and GSK3B, that regulate type 2 diabetes, non-insulin-dependent DM, and inflammatory diseases. These results successfully indicated the potential anti-diabetic mechanism of the active ingredients of *L. chinensis*.

## Introduction

Diabetes mellitus (DM) is a chronic inflammatory disease that seriously threatens human health, and is affected by the interaction between genetic and environmental factors (Yue et al., 2017a). DM is caused by a metabolic disorder of the endocrine system, and a long-term illness can lead to tissues and organs damage to the cardiovascular, endocrine, nervous, and urinary systems (Ge et al., 2017). Beta cell (β cell) failure plays an important role in the development of type 2 DM (T2DM). The dysregulation of metabolic and inflammatory process caused by the onset of DM contributes to the loss of islet function and impaired insulin secretion of β cells, and then affect the body’s immune system, leading to various complications of DM (Macdougall et al., 2019).

Traditional Chinese medicine (TCM) has been used for treating DM for more than 2000 years. Relevant studies have shown that TCM can significantly improve blood sugar and clinical indicators of diabetic patients, and effectively delay the progress of DM. (Tian et al., 2019). *Lobelia chinensis* (*L. chinensis* Lour.) is an annual dwarf herb, belonging to the family Campanulaceae, and is widely distributed in East Asian countries including China, Korea, and Japan (Yang et al., 2014). Modern pharmacology research indicates that the whole herb of *L. chinensis* contains a variety of alkaloids, which has medicinal functions of clearing heat-toxin, promoting diuresis, and diminishing inflammatory (Li et al., 2009). According to preliminary literature reports, *L. chinensis* contains flavonoids (Yang et al., 2014), terpenoids (Chen et al., 2014), lignans (Shibano et al., 2001), alkaloids (Kuo et al., 2011), and some other active ingredients. Although the active ingredients in *L. chinensis* are known, it is still unclear how the active ingredients act on target proteins and regulate signaling pathways to achieve the pharmacological effects of anti-inflammatory and anti-diabetic.

**Figure 1:**
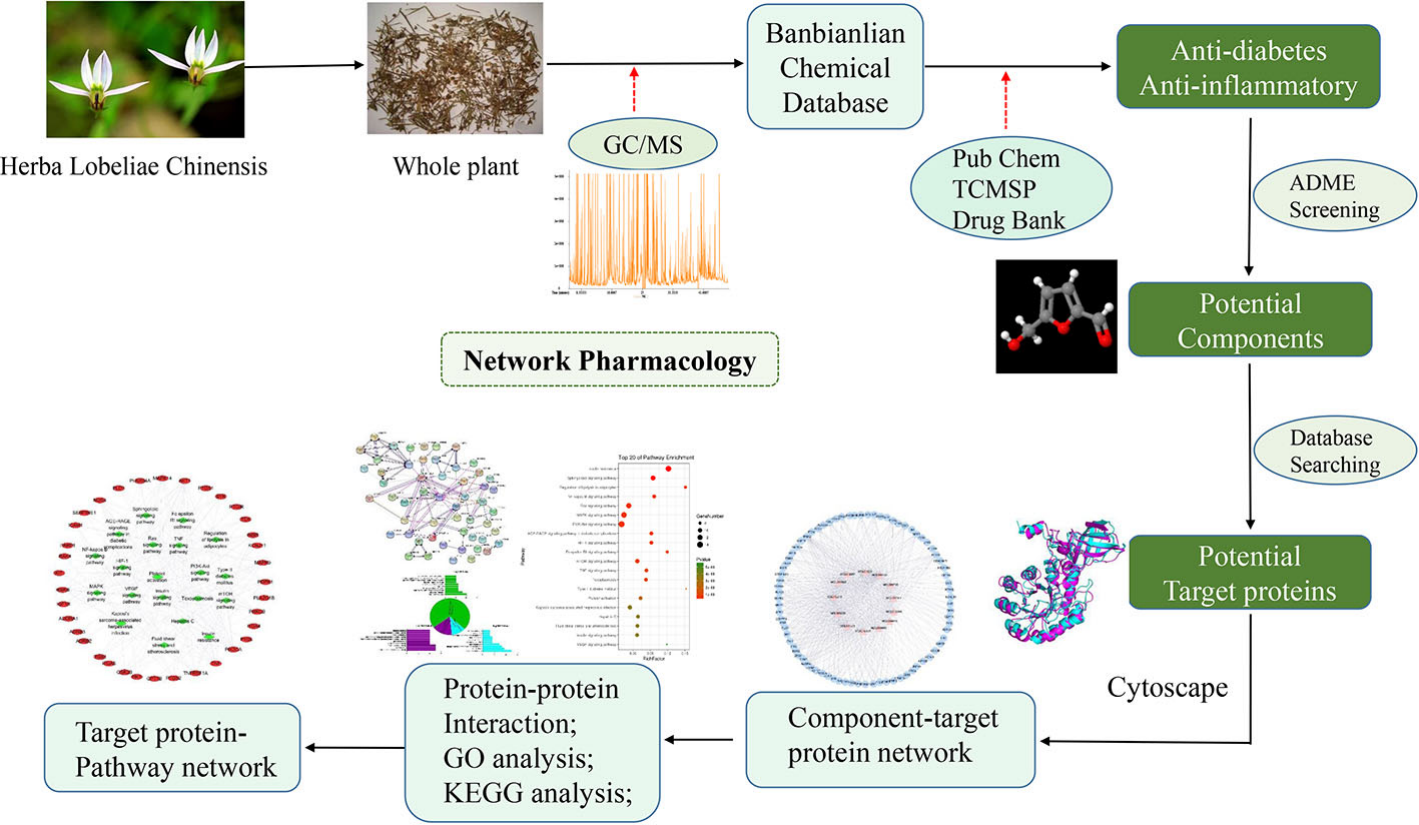
The flowchart of the network pharmacological analysis approach.

In order to comprehensively evaluate the pharmacological effects of TCM, network pharmacology has been introduced to explore the molecular mechanism of TCM in recent years (Hong et al., 2017a; Hong et al., 2017b). The development of systematic pharmacological studies on the relationship between biological processes and the treatment of TCM has attracted considerable interest. Therefore, by integrating systematic information with the overall characteristics of TCM to achieve a comprehensive analysis, and turning the idea of “one drug, one target and one disease” to multi-target combination (Pujol et al., 2010). Based on network interaction to study the basic biological knowledge of TCM can provide a deep insight or scientific evidence for the discovery of TCM, and help us to clarify the pharmacological mechanism of active ingredients of TCM at the level of biomolecule (Yang et al., 2018). Network pharmacology is gradually becoming a holistic and efficient tool to describe the complex interactions between drugs and biological systems including the human organs, diseases, metabolic pathways, and target proteins from a network perspective(Zhang et al., 2016). Combined with pharmacology and pharmacodynamics, it has been successfully applied to explain the mechanism of TCM at the molecular network level (Yue et al., 2017b). In this study, we first identified the active ingredients in *L. chinensis* by metabolomics analysis. Based on the pharmacokinetic and pharmacological data of components, we constructed a network pharmacological model of active ingredients, and systematically analyzed the potential anti-diabetes mechanism of active ingredients in *L. chinensis*. The detailed procedures can be seen in **Figure 1**.

## Materials and Methods

### Description of the Study Material

*L. chinensis* is a plant, commonly called Chinese lobelia, creeping lobelia, or Banbianlian, is a low-growing, tiny-leaved, herbaceous perennial that typically forms an attractive ground cover rising to only 2–3″ tall but spreading rapidly by creeping decumbent stems to 36″ wide. Creeping stems are clad with narrow, lanceolate, green leaves (to 3/4″ long). Pale pink to white, usually solitary flowers (1 1/4″across) bloom from the leaf axils from July to October. Each lobelia-like flower features two lanceolate to oblanceolate lateral lobes and three more prominent central elliptic lobes in a flattened plane. *L. chinensis* is 1 of the 50 fundamental herbs used in TCM (**Supplementary Figure 3**). Voucher specimen accession number NAS00276292 was deposited in Institute of Botany, Jiangsu Province and Chinese Academy of Sciences (**Supplementary Figure 1**).

### Determination of Active Ingredients From *L. chinensis*

*L. chinensis* was collected in Quanjiao, Anhui Province, and now is preserved in the Institute of Life Sciences, Jiangsu University. The whole plants were dried and ground to a fine powder in an electric grinder. All chemicals and reagents were analytical or HPLC grade. The 60 mg accurately weighted *L. chinensis* powder was transferred to a 2.0-ml Eppendorf tube. Two small steel balls were added to the tube, 360 μl of cold methanol and 40 μl of internal standard (0.3 mg/ml 2-chloro-L-phenylalanine, dissolved in methanol) were added to the tube, and then placed at −80°C for 2 min, ground at 60 Hz for 2 min, and sonicated for 30 min. After that, 200 μl of chloroform was added to the tube and the mixtures were mixed thoroughly at ambient temperature, then 400 μl of water was added. The samples were centrifuged at 12,000*g* for 10 min at 4°C. An aliquot of the 200 μl supernatant was transferred to a glass sampling vial for desiccation at room temperature. Eighty microliters of methoxyamine hydrochloride in pyridine (15 mg/ml) was subsequently added. The resultant mixture was vortexed vigorously for 2 min and incubated at 37°C for 90 min, then 80 μl of BSTFA (with 1% TMCS) and 20 μl n-hexane was added into the mixture, which was vortexed vigorously for 2 min and incubated at 70°C for 60 min. All the samples were placed at ambient temperature for 30 min before gas chromatography–mass spectrometry (GC-MS) analysis. And all the samples were tested in triplicate.

### GC-MS Experiments

The derivatized samples were analyzed on an Agilent 7890B gas chromatography system coupled to an Agilent 5977A MSD system (Agilent Technologies Inc., CA, USA). DB-5MS fused-silica capillary columns (30 m × 0.25 mm × 0.25 µm, Agilent J & W Scientific, Folsom, CA, USA) with highly pure helium (purity not less than 99.999%) at a constant ﬂow rate of 1.0 ml/min were utilized to separate the derivatives. The injector temperature was maintained at 260°C. Injection volume was 1 μl, and the split ratio was 10:1. The initial temperature of the column oven was 60°C, ramped to 125°C at a rate of 8°C/min, to 210°C at a rate of 4°C/min, to 270°C at a rate of 5°Cmin, to 305°C at a rate of 10°C/min, and finally held at 305°C for 3 min. The temperature of MS quadrupole and ion source (electron impact) was set to 150°C and 230°C, respectively. The collision energy was 70 eV. Scan mode was Full Scan mode (SCAN) and mass scan range was m/z 50–500.

### Chemical Ingredients Database Building and Screening

All of the constituent data of *L. chinensis* were obtained from Traditional Chinese Medicine Systems Pharmacology Database and Analysis Platform (TCMSP, http://lsp.nwu.edu.cn/tcmsp.php) (Ru et al., 2014). The active ingredients obtained from GC-MS and supplemented through a wide-scale text-mining method were input into TCMSP website to screen the chemical ingredients that interact with DM and inflammation. The effective ingredients of *L. chinensis* are mainly filtered by oral bioavailability (OB) and blood–brain barrier (BBB) permeability. OB is one of the most important pharmacokinetic parameters in the characteristics of absorption, distribution, metabolism, and excretion (ADME) characteristics of drugs, indicating the ratio of the oral drug to the oral dosage of the blood circulatory system (Fang et al., 2017). The ADME parameter-based virtual screening of the ingredients was utilized to further identify anti-diabetic ingredients using an OB threshold OB ≥ 30% and barrier permeability (BBB) ≥ −0.30 as parameters (Tsaioun et al., 2016). Meanwhile, the chemical information of these ingredients (structure, specification name, and CID number) for computational analysis were also collected according to the Pub Chem (https://pubchem.ncbi.nlm.nih.gov/) and Drug Bank (https://www.drugbank.ca/drugs).

### Ingredient–Target Network Construction

In order to identify the corresponding targets of the active ingredients of *L. chinensis*, a method combined with information integration and data mining was implemented. First of all, the active ingredients and interact targets were submitted to Cytoscape 3.2.1 software to construct an ingredient–target network to explore the pharmacological mechanism of the active ingredients of *L. chinensis*. The input data with Excel format file can be seen in the **Supplementary Tables 3** **and** **4**.

### Protein-Protein Interaction Network Construction

To explain the interaction between target proteins, the target proteins of related ingredients of *L. chinensis* were uploaded to STRING (http://string-db.org) online website to obtain the information of protein-protein interaction (PPI). The website generated a score for each protein mutual information. The higher the score, the higher the confidence in the interaction between target proteins. Therefore, we selected high confidence data >0.7 to ensure the reliability of this analysis. The obtained protein interaction data were imported into Cytoscape 3.2.1 software to construct a PPI protein interaction network.

### Gene Ontology and KEGG Enrichment Analysis of Target Proteins

To elucidate the role of target proteins that interact with the active ingredients of *L. chinensis* in gene function and signaling pathway, the Database for Annotation, Visualization and Integrated Discovery (DAVID, https://david.ncifcrf.gov/) v6.8 was used to analyze the Gene Ontology (GO) function and KEGG pathway enrichment of proteins involved in PPI network. The target proteins involved in the cellular components (CC), molecular function (MF), biological process (BP), and the pathways were also described.

### Ingredient–Target–Pathway Network Construction

After getting the interaction information of the active ingredient compounds, target proteins, and the pathways, the “Ingredient–Target–Pathway” network was established by Cystoscope 3.2.1 software. In the network, nodes represent components, targets, and pathways, edges represent the interaction of each other. Then a hypothesized schematic diagram of the target proteins involved in the pathways was drawn. Based on the network model map, the pathway of active ingredients and targets in the disease is initially explored to provide a preliminary theoretical basis for the design of subsequent targeted drugs.

## Results

### GC/MS Determination of Active Ingredients in *L. chinensis*

To demonstrate the ability of the GC/MS method to accurately separate and identify ingredients, the sample of *L. chinensis* powder was analyzed triple times by GC/MS. The results showed strong signals, high peak capacity, and well reproducibility. The typical total ion current (TIC) was shown in **Supplementary Figure 2**. The detected ingredients contained acacetin (4.78 mg/g), norlobelanine (6.05 mg/g), 5-hydroxymethylfurfural (1.26 mg/g), and other bioactive ingredients. The concentration of the active ingredients of *L. chinensis* are shown in **Table 1**. A total of 208 chemical ingredients were identified and shown in **Supplementary Table 1**.

**Table 1:** The concentration of the active ingredients of *Lobelia chinensis*.

MOL ID	Ingredients	Concentration
MOL012231	Leptodactylone	23.58 mg/g
MOL012207	Lobelanidine	12.62 mg/g
MOL001689	Acacetin	4.78 mg/g
MOL012216	Norlobelanine	6.05 mg/g
MOL001999	Scoparone	0.13 mg/g
MOL005928	Isoferulic acid	22.77 mg/g
MOL012209	2-[(2R,6S)-6-[(2R)-2-hydroxy-2-phenylethyl]-1-methylpiperidin-2-yl]-1-phenylethanone (Lobelin)	46.60 mg/g
MOL009009	(+)-Medioresinol	0.84 mg/g
MOL004678	Limetin	2.51 mg/g
MOL012208	Lobelanine	27.88 mg/g
MOL002341	Hesperetin	3.27 mg/g
MOL000748	5-Hydroxymethylfurfural (HMF)	1.26 mg/g
MOL000103	4-Oxoniobenzoate (PHB)	5.49 mg/g

### Screening of Effective Anti-Diabetic Ingredients in *L. chinensis*

Early assessment of the ADME properties of candidate active ingredients has become an important process in modern drug discovery. And the correct use of ADME identifies candidates that are more likely to have good pharmacokinetic properties. Currently, OB and BBB are considered as a key parameter in drugs discovery. TCMs are often administered orally, and its OB is determined by body absorption, distribution, and liver metabolism. It is important to identify the active ingredients of medicinal herbs. In this research, OB and BBB indicators were used to screen for *L. chinensis* ingredients with favorable pharmacokinetic properties. Therefore, all chemicals that meet the screening criteria: OB (> 30%) and BBB (>-0.3) are considered as candidate ingredients. Twenty-three differentiated ingredients with effective pharmacological activities were identified after screening ADME parameters (**Table 2**).

**Table 2:** Ingredients information of *Lobelia chinensis*.

MOL ID	Name	Molecular Formula	CAS No.	MW (g/mol)	OB%	BBB	DL	HL	Structural Formula
MOL012207	Lobelanidine	N/A	552-72-7	339.52	60.53	0.31	0.32	5.78	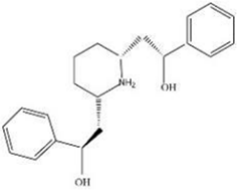
MOL012208	Lobelanine	C22H25NO2	579-21-5	335.48	54.13	0.46	0.32	31.96	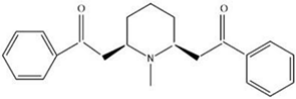
MOL000103	4-Oxoniobenzoate (PHB)	C7H6O3	99-96-7	138.13	30.15	0.21	0.03	11.77	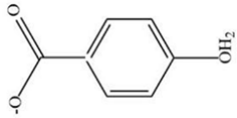
MOL012209	2-[(2R,6S)-6-[(2R)-2-hydroxy-2-phenylethyl]-1-methylpiperidin-2-yl]-1-phenylethanone (Lobelin)	C22H27NO2	90-69-7	337.50	45.53	0.22	0.32	19.19	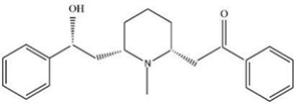
MOL011678	(3S,8S,9S,10R,13R,14S,17R)-17-[(1S,4R)-4-ethyl-1,5-dimethylhexyl]-10,13-dimethyl-2,3,4,7,8,9,11,12,14,15,16,17-dodecahydro-1H-cyclopenta[a]phenanthren-3-ol	C29H50O	474-58-8	414.79	36.91	1.15	0.75	4.93	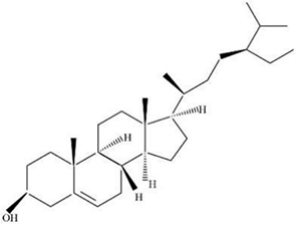
MOL012215	Propanol (POL)	C3H8O	71-23-8	60.11	72.69	0.97	0.00	11.62	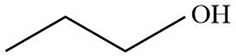
MOL012216	Norlobelanine	C21H23NO2	6035-31-0	321.45	64.08	0.33	0.30	35.32	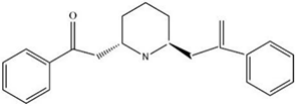
MOL012217	Phytenal	N/A	13754-69-3	294.58	35.22	1.41	0.14	3.96	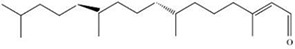
MOL012221	Trans-10-ethyl-8-methyl-lobelidiol	N/A	N/A	229.41	33.93	-0.04	0.07	2.73	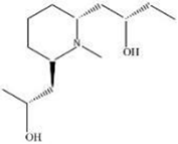
MOL012223	Trans-N-methyl-2,6-bis(2-hydroxybutyl)-△3-piperideine	N/A	N/A	243.44	42.40	-0.19	0.08	4.73	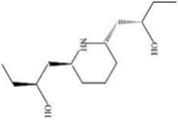
MOL012231	Leptodactylone	C11H10O5	61899-44-3	222.21	37.56	0.22	0.10	2.68	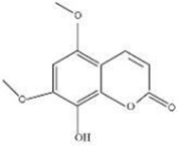
MOL001999	Scoparone	C11H10O4	120-08-1	206.21	74.75	0.46	0.09	0.73	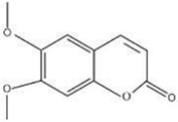
MOL002341	Hesperetin	C16H14O6	520-33-2	302.30	70.31	-0.25	0.27	15.78	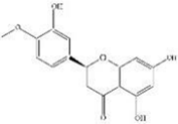
MOL004678	Limetin	C11H10O4	487-06-9	206.21	36.63	0.47	0.09	1.33	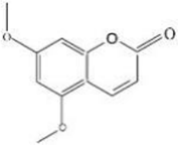
MOL005928	Isoferulic acid	C10H10O4	537-73-5	194.20	50.83	0.01	0.06	2.45	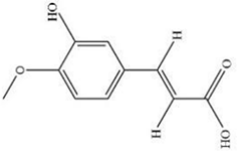
MOL007170	Cirsiumaldehyde	C12H10O5	7389-38-0	234.22	41.38	0.03	0.11	7.81	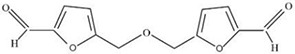
MOL000748	5-Hydroxymethylfurfural (HMF)	C6H6O3	76330-16-0	126.12	45.07	-0.27	0.02	11.73	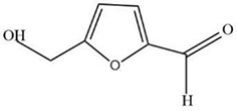
MOL009009	(+)-Medioresinol	N/A	40957-99-1	388.45	87.19	-0.29	0.62	1.39	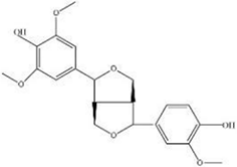
MOL012232	5-Hydroxy-6,7-dimethoxycoumarin	C11H10O5	28449-62-9	222.21	67.31	0.20	0.10	1.83	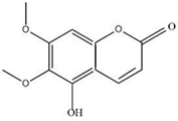
MOL009653	Cycloeucalenol	N/A	469-39-6	426.80	39.73	1.04	0.79	5.01	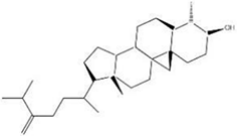
MOL001504	(E,7R,11S)-3,7,11,15-tetramethylhexadec-2-en-1-ol	C20H40O	150-86-7	296.60	33.82	0.90	0.13	3.46	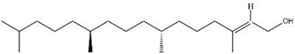
MOL001689	Acacetin	C16H12O5	480-44-4	284.28	34.97	-0.05	0.24	17.25	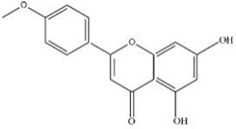
MOL004688	Cumic acid	C10H12O2	536-66-3	164.22	45.78	1.10	0.04	-3.13	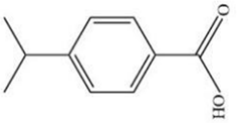

### Interactions Analysis of Active Ingredients and Target Proteins

**Figure 2:**
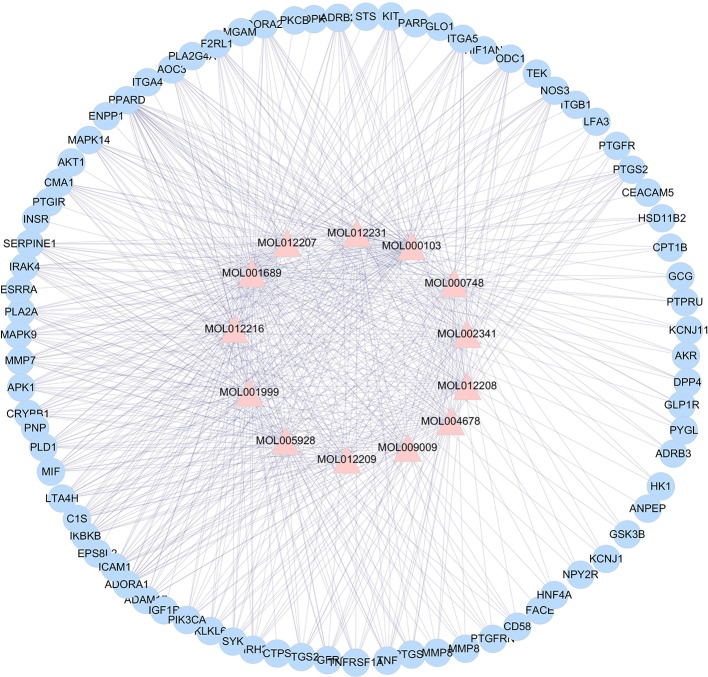
*Lobelia chinensis* ingredients–target interaction network. The pink triangle represent the 13 bioactive ingredients, and the blue circle represent the 80 target proteins.

According to the analysis, the active ingredients of *L. chinensis* have good pharmacological effects on anti-inflammatory and anti-diabetic in a synergistic way. To further study the mechanism of anti-diabetes, it is of paramount important to understand the target proteins on which these ingredients act. With the development of network pharmacology, it provides an effective tool for the study of TCM pharmacology. Using TCMSP, the active ingredients of *L. chinensis* and the target proteins related to inflammation and DM were searched. Cytoscape 3.2.1 was used to analyze the interaction between 23 active ingredients and target proteins. The results are shown in **Figure 2**. The analysis ingredients–targets network consisted a total of 93 nodes and 581 edges, of which 13 are anti-inflammatory and anti-diabetic active ingredients, and the other 80 are target protein nodes. The result shows that MOL001689 (acacetin), MOL012216 (norlobelanine), MOL000748 (5-hydroxymethylfurfural) have more target proteins play a key role in the interaction network, which may be the key ingredients or target proteins that play an anti-inflammation and anti-diabetic role in *L. chinensis*. **Supplementary Table 2** lists the ingredients and the related target proteins. Based on the number of ingredients–targets relationships, the number of target proteins acting on average per ingredients is about 6.15. These results indicate that muti-ingredients muti-targets interactions occur in *L. chinensis*.

### Construction and Analysis of Target Proteins PPI Network

**Figure 3:**
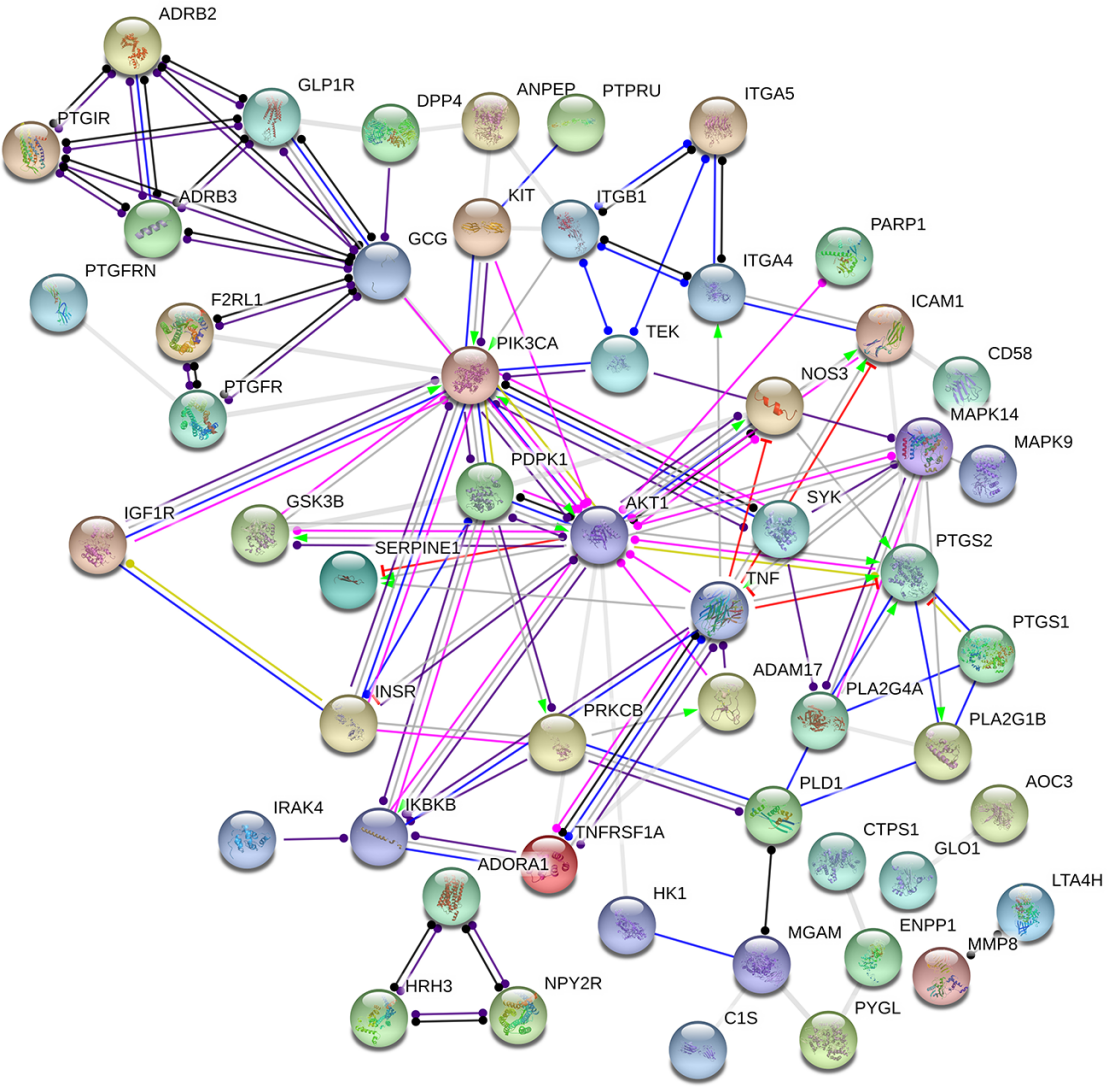
Protein–protein interaction (PPI) network analysis.

The target proteins that act with its corresponding ingredients were submitted to STRING version 10.5 (http://string-db.org/) for PPI network construction, and high confidence of protein interaction data with a score >0.7 was selected (**Figure 3**). The results include a total of 75 nodes, 107edges, of which nodes represent the target proteins and the edges represent the interactions between the proteins. In this network interaction, the larger the degree, the stronger relationship between the proteins corresponding to the node in this network, which indicates that the target proteins play a key role in the whole interaction network, which is the important target protein. **Figure 3** shows that PIK3CA, PDPK1, AKT1, and TNF are centrally located in the PPI network, indicating that these proteins are involved in the pathogenesis of DM and inflammation.

### GO Analysis of Target Proteins

**Figure 4:**
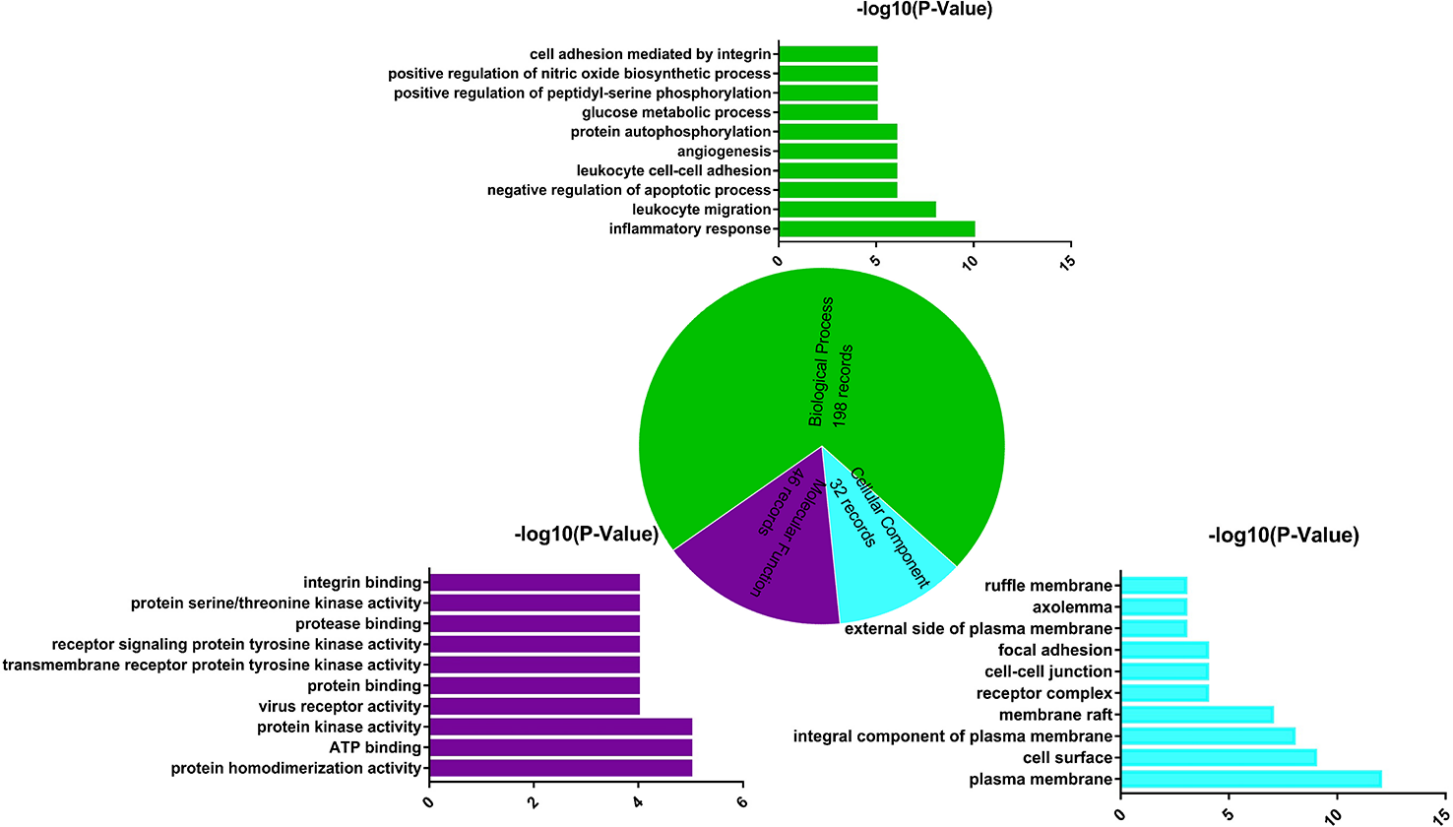
Gene Ontology (GO) enrichment analysis of the target proteins. Biological process (green), molecular function (purple), and cellular component (blue) accounted for 71.47%, 16.67%, and 11.59%, respectively.

GO enrichment analysis of target proteins that act with its corresponding ingredients was performed by DAVID. The top 10 significantly enriched terms in BP, MF, and CC categories were selected, according to P < 0.05, P-values were corrected using the Benjamini–Hochberg procedure. As shown in **Figure 4**, BP (198 records), MF (40 records), and CC (32 records) accounted for 71.74%, 16.67%, and 11.59%, respectively. In the BP category, the target proteins were mainly involved in inflammatory response, participate in leukocyte migration, and glucose metabolic process. In the MF category, the target proteins were mainly involved in protein homodimerization activity and ATP binding. In the CC category, the target proteins were classified into plasma membrane and cell surface. The GO enrichment analysis results showed that the active ingredients of *L. chinensis* could bind kinase in cell membrane and plasma membrane in the process of inflammation, so as to exert anti-inflammatory and anti-diabetic potential.

### KEGG Classification of Target Proteins

**Figure 5:**
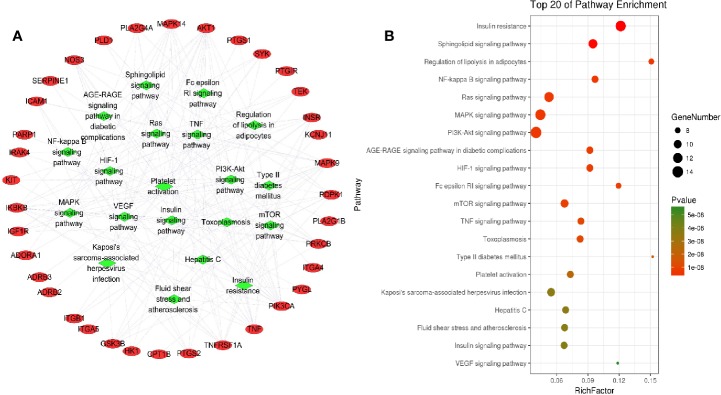
Targets–pathway interaction analysis **(A)** and KEGG analysis of target proteins **(B)**. The red ellipse represent the target proteins, and the green diamond represent the pathways.

To further clarify the relationship between target proteins and the pathways, we constructed a target–pathway interaction network using the data extracted from DAVID database (**Figure 5A**), and the top 20 pathways involving 33 target proteins were screened according to the KEGG analysis with BH-corrected P-values < 0.05 (**Figure 5B**). The results showed that these target proteins were mainly involved in insulin resistance, sphingolipid signaling pathway, regulation of lipolysis in adipolysis, NF-kappa B signaling pathway, MAPK signaling pathway, PI3K–AKT signaling pathway, and so on. The target proteins involved in insulin resistance included RAC-alpha serine/threonine-protein kinase (AKT1), carnitine palmitoyltransferase 1B (CPT1B), glycogen synthase kinase 3 beta (GSK3B), inhibitor of nuclear factor kappa B kinase subunit beta (IKBKB), INSR, mitogen-activated protein kinase 9 (MAPK9), nitric oxide synthase 3 (NOS3), phosphoinositide-dependent kinase-1 (PDPK1), phosphatidylinositol 4,5-bisphosphate 3-kinase catalytic subunit alpha isoform (PIK3CA), protein kinase C beta (PRKCB), glycogen phosphorylase (PYGL), tumor necrosis factor (TNF), and tumor necrosis factor receptor superfamily member 1A (TNFRSF1A); the target proteins involved in sphingolipid signaling pathway were adenosine A1 receptor (ADORA1), AKT1, mitogen-activated protein kinase 14 (MAPK14), MAPK9, NOS3, PDPK1, PIK3CA, phospholipase D1 (PLD1), protein kinase C beta (PRKCB), TNF, and TNFRSF1A; the target proteins involved in MAPK signaling pathway were AKT1, insulin-like growth factor 1 receptor (IGF1R), IKBKB, INSR, interleukin-1 receptor-associated kinase 4 (IRAK4), mast/stem cell growth factor receptor Kit (KIT), MAPK14, MAPK9, phospholipase A2 group IVA (PLA2G4A), PRKCB, angiopoietin-1 receptor (TEK), TNF, and TNFRSF1A. Therefore, there are multiple target proteins in one pathway, and the same target protein exists in multiple pathways. Essentially, a pathway involving multiple target proteins is more important than the interaction between one target protein and multiple pathways. These results suggest that the effective pharmacological active ingredients in *L. chinensis* may act on these signaling pathways to alleviate inflammation, including some chronic inflammation, such as type 2 diabetes and other diseases.

### Network Analysis of Target Proteins Involved in Signaling Pathways

**Figure 6:**
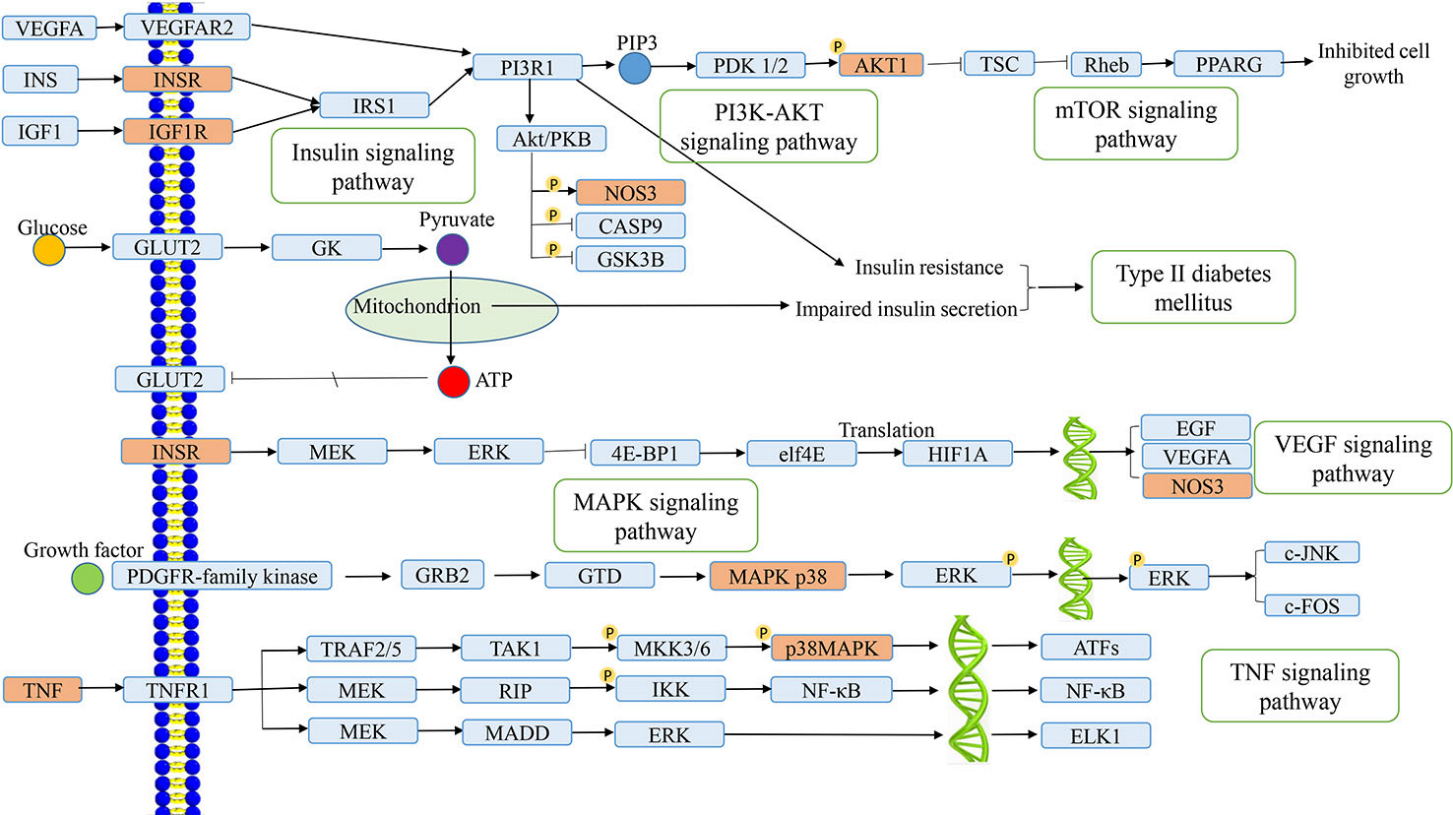
Distribution of the target proteins of *Lobelia chinensis* on the predicted pathway. The orange nodes are potential target proteins of *L. chinensis*, while the blue nodes are relevant targets in the pathway.

As an important part of systemic pharmacology, signaling pathways can link receptor–ligand interactions with pharmacodynamics pathways. All target proteins interacting with the active ingredients of *L. chinensis* were located in the top 20 KEGG pathways and then generated a target–pathway signaling network. Among them, there are lots of target proteins involved in the PI3K–AKT signaling pathway, insulin signaling pathway, and TNF signaling pathway, which play an explicit role in maintaining insulin secretion, glucose homeostasis, and inflammation (Taniguchi et al., 2006). TNF is associated with a variety of inflammatory, infectious and malignant diseases (Bradley, 2008). In addition, the vascular endothelial growth factor signaling pathway that involving many target proteins, such as INSR, NOS3, PTGS2, and vascular endothelial growth factor A (VEGFA), plays a key regulatory role in diabetic retinopathy (Antonetti et al., 2012). VEGF can cause injury of vascular endothelial cells (ECs), inflammation, and oxidative stress, which can lead to an abnormal metabolism of blood glucose caused by diabetes, and also cause the insulin secretion from islet cells due to elevation of blood glucose, that aggravates the symptoms of insulin resistance (Zhang et al., 2006). mTOR is a serine/threonine-protein kinase whose signal transduction regulates protein synthesis and cell growth, especially hypertrophy. Therefore, activation of the mTOR signaling pathway is a potential reason for early renal hypertrophy in DM (Sakaguchi et al., 2006). Interestingly, after network construction, we also found that active ingredients can play a therapeutic role in type 2 diabetes by activating the VEGFA to regulate the expression of PI3R1 and decreasing insulin resistance. As shown in **Figure 6**, the anti-inflammatory and anti-diabetic active ingredients present in *L. chinensis*, which can synergize with multiple target proteins in these pathways to form a multi-ingredients-multi-targets-multi-pathways mechanism.

## Discussion

Medicinal plants have a long history in the treatment of chronic inflammatory diseases such as DM and various complications. In the pharmacological study of active ingredients, research methods based on network interaction are expected to highlight the understanding of drug action across multiple layers of information. The network model of active ingredients and target proteins is an important way to characterize the pharmacological mechanism of active ingredients, which can provide a theoretical basis for future drug development and design (Boezio et al., 2017). The research ideas of network pharmacology provide a unique and innovative way for the study of active ingredients in TCM, in order to understand the mechanism of the multi-ingredients-multi-targets mode of TCM (Ge et al., 2018a).

In this study, 208 metabolites of active ingredients in *L. chinensis* were determined by GC/MS metabonomics analysis. Among them, 23 active ingredients such as lobelanidine, 5-hydroxymethylfural, acacetin, and hesperetin possess the pharmacological activities (**Table 2**), of which 13 ingredients have anti-inflammatory and anti-diabetic effects (**Figure 2**). Subsequently, according to the analysis of network pharmacology, we found that these ingredient compounds which have therapeutic effects on inflammation and diabetes-related symptoms can treat or alleviate the symptoms of inflammation and diabetes, through the role of related target proteins in metabolic pathways caused by the occurrence of diseases. GO and KEGG analysis results showed that the target proteins involved in main pathways were inflammation, non-insulin dependent pathway, MAPK signaling pathway, PI3K–AKT signaling pathway, TNF signaling pathway, and DM signaling pathway (**Figure 5**). The systematic pharmacological analysis showed that the active ingredients of 5-hydroxymethylfural and acacetin in *L. chinensis* could stimulate insulin secretion, improve insulin resistance, and promote glucose utilization by acting on GSK3B, MAPK, INR, and dipeptidyl peptidase-4 (DPP4). Meanwhile, they could also regulate inflammatory cytokines, promote endocrine and metabolism, and achieve the therapeutic effect in the treatment of diabetes.

GSK3B is a serine-threonine kinase in glycogen synthase subfamily, and it is also a negative regulator of glycometabolism balance that participates in energy metabolism, inflammatory response, endoplasmic reticulum stress, mitochondrial dysfunction, and apoptotic pathway (MacAulay and Woodgett, 2008). Selective inhibition of GSK3B in insulin resistance pathway can improve insulin-stimulated glucose transport activity, which may be due to enhanced insulin signal transduction and GLUT-4 translocation (Henriksen and Dokken, 2006). Therefore, inhibition of GSK3B may have the potential to treat DM and insulin resistance. DPP4 is capable of rapidly degrading endogenous GLP-1, which plays a crucial role in glucose metabolism (Barnett, 2006). At present, GLP-1 is the only known protein to reduce blood glucose levels by increasing insulin secretion (MacDonald et al., 2002). The hypoglycemic mechanism of two diabetic drugs such as sitagliptin and vildagliptin is also based on DPP4 inhibitors (Karasik et al., 2008; Forst and Bramlage, 2014). Therefore, it is important to evaluate the inhibitory effect of active ingredients of *L. chinensis* on DPP-4.

According to the results of pathway analysis, mitogen-activated protein kinases (MAPKs), including extracellular signal-regulated kinase (ERK), c-Jun N-terminal kinase (JNK), and p38 MAPK, may also play a crucial role in regulating diabetes and inflammation through the MAPK signaling pathway. The MAPKs are extracellular signal-regulated protein kinases, increasing cellular proliferation and involving in the cell survival regulation (Chen and Jiang, 2018). MAPK can also be inactivated by protein phosphatases, which counteract many kinase-driven intracellular signaling pathways through dephosphorylating key proteins (Perrotti and Neviani, 2013). The liver is the main important organ to control metabolism in the human body (Ge et al., 2018b). MAPKs can regulate inflammatory response along with a wide range of cellular processes including cell metabolism, proliferation, motility, apoptosis, survival, differentiation, and survival in physiological and pathological processes (Li et al., 2020). Previous studies have shown that p38 MAPK controls cellular responses to cytokines and stress (Zielinski and Krueger, 2012). Thus, MAPK p38 inhibitors inhibit the production of many proinflammatory cytokines, including IL-1β and TNF-α, and inducible nitric oxide synthase (iNOS) (Sun et al., 2017). These findings further confirm that the pharmacological activation of MAPK may be mediated by signal transduction, metabolism, and gene expression, while changes in liver metabolism may lead to the occurrence of insulin resistance, type 2 diabetes, and obesity. Therefore, we think that MAPK may be associated with metabolic diseases, which also provides a new strategy for the treatment of metabolic diseases such as type 2 diabetes and obesity.

Additionally, TNF signaling pathway can induce multiple cascade reactions, including activation of the transcription factor NF-kappa B and programmed cell death (Van Antwerp et al., 1996). TNF activates the pro-inflammatory function of vascular ECs by binding to cytokines such as TNFRSF1A/TNFR1 and TNFRSF1B/TNFBR expressed on the cell surface and is a potent pyrogen (Wang et al., 2003). It is mainly secreted by macrophages and can induce cell death in some cancer cell lines (Wang et al., 1996). Previous researches on TNF signaling have indicated that TNF signaling pathway is a negative feedback mechanism, and the activation of NF-kappa B inhibits the signal of cell death (Ge et al., 2018a). Mavrogonatou and Ji *et al*. have shown that TNF-alpha is an inflammatory mediator, and its overexpression can lead to the rapid activation of p38MAPK under the external or internal stimulation, which promotes the production of IL-6 and induces the body’s inflammatory response (Ji et al., 2016; Mavrogonatou et al., 2018).

T2DM is caused by insulin resistance and loss of islet beta-cell function. Insulin signaling occurs through the INSR, which is selectively spliced into two isomers: INSR-A and INSR-B (Besic et al., 2015). Malakar *et al*. showed that insulin could regulate selective splicing of INSR through Ras-MAPK/ERK signaling pathway and that INSR-B had a protective effect in β cells, while the expression of INSR-A made β cells sensitive to programmed cell death (Malakar et al., 2016). Previous studies have also shown that insulin signal transduction pathway plays a crucial role in the pathogenesis of diabetes, which is of great significance for the study of insulin signal transduction in type 2 diabetes (Ge et al., 2018a). Insulin binding to INSR on cell membranes will induce signal transduction, and the insulin receptor substrate (IRS) plays a key role in insulin signaling system (Cai et al., 2016). It mediates multidirectional cell signal transduction effects such as IRS and insulin-like growth factor (IGF), thereby exercising the connection and functional regulation with other cytokines (Moon et al., 2015).

This enrichment pathway analysis result indicates that insulins can bind to INSR on the cell membrane, and affect the occurrence of diabetes and inflammation through PI3K–AKT signal transduction pathway. After release of insulin into the blood, insulin could bind to insulin receptor (INSR) on the surface of liver cell membrane and then activates insulin receptor phosphorylates tyrosine site of IRS-1/2, regulating the PI3K/AKT signal pathway, of which IRS2 is the main target in the insulin metabolism effects in liver (Wang et al., 2013). The activated PI3K can catalyze 4, 5-2 phosphatidyl inositol phosphate (PIP2) and generate PIP3, which may act the second messenger to activate AKT, and the activated AKT has an effect on the biological metabolism by regulating downstream molecules (Dai et al., 2016). Therefore, any abnormality in the PI3K–AKT signaling pathway will affect insulin signal transduction, thereby promoting the occurrence and development of insulin resistance and type 2 diabetes.

In summary, the pathogenesis of DM is more complex, and the signal pathways are cross-linked and mutually modulated, presenting a multi-pathway and multi-target pattern. The active ingredients of *L. chinensis* can regulate target proteins involved in signal pathways and play a role in stabilizing signal pathway, revealing the multi-ingredient–multi-target–multi-pathway action mode of TCM. Deep analysis of anti-diabetic and anti-inflammatory pharmacological effects of *L. chinensis*, as well as the target and pathway acting with the active ingredients still need to be further validated.

## Data Availability Statement

All datasets generated for this study are included in the article/**Supplementary Material**.

## Author Contributions

QG is the major contributor to this manuscript. QG conducted the analytical part, wrote the first version of the manuscript, and finalized the manuscript. LC and YY downloaded the reference and processed the graph and the table in the manuscript. LL, FF, PL, and SM collected the data. KC (corresponding author) and QY (corresponding author) conceived and coordinated the study, and critically evaluated the data. All authors read and approved the final manuscript.

## Funding

This work was supported by the National Natural Science Foundation of China (No. 31861143051, 31872425, 31802140, and 81672913) and the Maternal and Child Health Research Project of Jiangsu Province (F201604).

## Conflict of Interest

The authors declare that the research was conducted in the absence of any commercial or financial relationships that could be construed as a potential conflict of interest.
