# Supplementary material for: Network Pharmacology-Based Dissection of the Anti-diabetic Mechanism of Lobelia chinensis
Source: Front Pharmacol. 2020 Mar 20;11:347. doi: 10.3389/fphar.2020.00347 (PMC7099657; doi:10.3389/fphar.2020.00347)
Supplement: Supplementary file 1 [file DataSheet_1.docx]

**Supplementary Tables and Figures**

**Supplementary Table S1. A total of 208 chemical ingredients of *Lobelia chinensis***

| **Metabolites** | **Quant Mass** | **RT(min)** | **Concentration (mg/g)** |
| --- | --- | --- | --- |
| phenylacetaldehyde | 193 | 5.14419 | 0.077620523 |
| 2-ketobutyric acid | 89 | 5.16494 | 2.716774002 |
| 1-Hydroxyanthraquinone | 282 | 5.172725 | 0.092878919 |
| Dithioerythritol | 221 | 5.20645 | 0.669705492 |
| glutamine | 155 | 5.25315 | 0.377618581 |
| 2'-Deoxycytidine 5'-triphosphate degr prod | 58 | 5.2669833 | 4.970897248 |
| asparagine | 159 | 5.32233 | 48.76664794 |
| p-benzoquinone | 121 | 5.39324 | 19.73247792 |
| 3-hydroxy-L-proline | 86 | 5.4157233 | 8.246110612 |
| lactic acid | 117 | 5.458095 | 22.72107197 |
| Citraconic acid degr1 | 89 | 5.50739 | 1.106615886 |
| dibenzofuran | 56 | 5.5333333 | 11.02824173 |
| Analyte 15 | 82 | 5.65094 | 0.189279185 |
| Benzoylformic acid | 119 | 5.7581733 | 0.106664038 |
| Leptodactylone | 102 | 5.97956 | 23.58194579 |
| Nicotinoylglycine | 295 | 6.05912 | 0.610430115 |
| 2-hydroxypyridine | 152 | 6.08506 | 9.787997876 |
| Prostaglandin E2 | 225 | 6.163755 | 0.09858246 |
| Mono(2-ethylhexyl)phthalate | 221 | 6.1767267 | 0.245238412 |
| farnesol | 123 | 6.22342 | 0.294842866 |
| Pyruvic acid | 174 | 6.2805 | 0.381187195 |
| Lobelanidine | 174 | 6.39984 | 12.62280575 |
| oxalic acid | 190 | 6.52955 | 0.10931504 |
| Cumic Acid | 221 | 6.59009 | 0.930960655 |
| Dehydroascorbic Acid | 173 | 6.73191 | 0.462090196 |
| glycolic acid | 177 | 6.74575 | 4.477772656 |
| Acacetin | 147 | 6.94291 | 4.780304612 |
| 5-Methoxytryptamine | 174 | 7.07263 | 34.16279748 |
| Maleamate | 151 | 7.10549 | 0.669342626 |
| alanine | 116 | 7.21791 | 270.6635726 |
| hydroxylamine | 249 | 7.4358333 | 0.409221915 |
| 1-Methylhydantoin | 258 | 7.48253 | 43.28928565 |
| 5,6-dihydrouracil | 171 | 7.58803 | 0.890317945 |
| 2-hydroxybutanoic acid | 131 | 7.6243533 | 0.78887573 |
| Methylmalonic acid | 218 | 7.69008 | 0.274541065 |
| 2-aminoethanethiol | 188 | 7.7385033 | 1.617115077 |
| 2-Furoic Acid | 125 | 7.80596 | 0.592818584 |
| Pipecolinic acid | 156 | 7.818925 | 1.013267678 |
| 3-Hydroxypyridine | 152 | 7.8509233 | 0.517886247 |
| Aminooxyacetic acid | 86 | 7.8786 | 3.76184874 |
| 3-Hydroxypropionic acid | 177 | 7.9547 | 1.999880355 |
| Norleucine | 86 | 8.17954 | 107.1727401 |
| 3-hydroxybutyric acid | 233 | 8.2331533 | 0.452073905 |
| Gallic acid | 282 | 8.36979 | 3.682017377 |
| thymidine | 70 | 8.514205 | 207.0971782 |
| Norlobelanine | 241 | 8.52199 | 6.050845762 |
| trans-3,5-Dimethoxy-4-hydroxycinnamaldehyde | 225 | 8.6084667 | 0.037198718 |
| succinate semialdehyde | 113 | 8.7451 | 0.076479121 |
| Scoparone | 171 | 8.778825 | 0.134845498 |
| Isoferulic acid | 147 | 9.00799 | 22.777472 |
| valine | 144 | 9.22073 | 249.4399294 |
| Carnitine | 227 | 9.3072 | 0.160538767 |
| Lyxose | 103 | 9.3833 | 1.293924282 |
| Lobelin | 217 | 9.49226 | 46.60207974 |
| 2-ketoadipate | 100 | 9.79839 | 0.231686889 |
| urea | 179 | 9.824335 | 3.395713446 |
| Ethanolamine | 174 | 10.210033 | 159.2485533 |
| N-Acetyl-L-leucine | 84 | 10.2308 | 118.2122784 |
| phosphate | 299 | 10.293033 | 1005.638588 |
| glycerol | 205 | 10.3657 | 362.0298337 |
| octanal | 58 | 10.46255 | 0.38081547 |
| (+)-Medioresinol carboxylic acid | 231 | 10.635533 | 0.848195096 |
| Adipamide | 183 | 10.6545 | 0.19218746 |
| 2-Deoxyerythritol | 117 | 10.682233 | 1.936660485 |
| Isoleucine | 158 | 10.749633 | 140.1221782 |
| L-Allothreonine | 117 | 10.7635 | 6.360332873 |
| nicotinic acid | 180 | 10.8102 | 3.46878353 |
| proline | 142 | 10.834367 | 131.8577315 |
| maleic acid | 245 | 10.962333 | 0.339694961 |
| glycine | 174 | 11.014233 | 70.88597518 |
| 2,3-Dihydroxypyridine | 240 | 11.176 | 0.508569768 |
| succinic acid | 247 | 11.206233 | 16.77818321 |
| conduritol b epoxide | 219 | 11.272 | 0.110873379 |
| 4-nitrophenol | 196 | 11.4172 | 0.322623504 |
| D-Glyceric acid | 295 | 11.529667 | 0.866046766 |
| Limetin | 241 | 11.685367 | 2.516176805 |
| Citraconic acid | 355 | 11.873867 | 0.003628077 |
| fumaric acid | 245 | 12.032933 | 17.72817981 |
| serine | 204 | 12.2111 | 378.7731121 |
| oxoproline | 156 | 12.282067 | 563.8325346 |
| 3-Cyanoalanine | 141 | 12.4394 | 3.174592508 |
| 4-hydroxybutyrate | 117 | 12.4913 | 2.124043599 |
| D-erythronolactone | 247 | 12.484367 | 1.172056984 |
| 3,4-Dihydroxypyridine | 241 | 12.733467 | 0.066970981 |
| threonine | 219 | 12.788733 | 73.71534529 |
| 6-hydroxy caproic acid | 261 | 12.9972 | 0.060278533 |
| Glutaric Acid | 261 | 13.28 | 0.237411951 |
| Diglycerol | 103 | 13.4201 | 13.58856525 |
| 5-Aminovaleric acid | 174 | 13.594767 | 74.31680199 |
| Lobelanine | 248 | 13.7833 | 27.88972269 |
| 2-Deoxytetronic acid | 233 | 13.9251 | 0.321648309 |
| Dodecanol | 243 | 14.025367 | 2.591876982 |
| Hesperetin | 218 | 14.3255 | 3.272603407 |
| thymine | 256 | 14.663567 | 0.380540756 |
| Dihydroxyacetone | 173 | 14.807133 | 0.850485768 |
| Citramalic acid | 247 | 14.824467 | 2.142842983 |
| N-alpha-Acetyl-L-ornithine | 241 | 14.976667 | 0.01529912 |
| L-Malic acid | 245 | 15.34245 | 412.17767 |
| putrescine | 174 | 15.6633 | 65.75024696 |
| Melatonin | 246 | 15.9495 | 0.594601602 |
| aspartic acid | 232 | 16.109533 | 97.33329398 |
| 4-aminobutyric acid | 174 | 16.317033 | 1078.168083 |
| L-glutamic acid | 84 | 16.403533 | 20.94542276 |
| Purine riboside | 243 | 16.6733 | 0.185584491 |
| Threonic acid | 220 | 16.687167 | 7.489460103 |
| Acetol | 219 | 16.903333 | 2.238486671 |
| glutamic acid | 246 | 16.9639 | 178.4168097 |
| tartaric acid | 147 | 17.008 | 3.788896302 |
| 3-hydroxybenzoic acid | 267 | 17.240633 | 0.625008312 |
| threo-beta-hyrdoxyaspartate | 220 | 17.366 | 1.573869768 |
| 2-hydroxy-3-isopropylbutanedioic acid | 261 | 17.4257 | 0.072917996 |
| 5-Hydroxymethylfurfural | 247 | 18.1936 | 1.263696062 |
| phenylalanine | 192 | 18.7125 | 61.81412067 |
| Lyxonic acid, 1,4-lactone | 217 | 18.7877 | 4.180061307 |
| 4-Hydroxybenzoic acid | 225 | 18.809333 | 0.213090735 |
| 2,6-Diaminopimelic acid | 200 | 18.8214 | 2.181476569 |
| xylose | 103 | 19.3559 | 10.05842323 |
| Erythrose | 205 | 20.14975 | 8.463129491 |
| ribose | 103 | 20.1826 | 21.70679514 |
| flavin adenine degrad product | 231 | 20.526767 | 0.551948383 |
| Levoglucosan | 204 | 20.7845 | 2.627984756 |
| Sedoheptulose | 204 | 21.00845 | 10.69943154 |
| 4-Oxoniobenzoate | 117 | 21.1442 | 5.493678762 |
| D-Arabitol | 217 | 21.268767 | 63.41528317 |
| Glucoheptonic acid | 205 | 21.7305 | 25.20597391 |
| 3,6-Anhydro-D-galactose | 231 | 21.945 | 1.801698131 |
| L-cysteine | 211 | 22.088533 | 0.788982955 |
| tyrosine | 218 | 22.213067 | 81.31637496 |
| 4-hydroxy-3-methoxybenzoic acid; | 282 | 22.2926 | 0.268591645 |
| Galactonic acid | 217 | 22.3445 | 33.59893004 |
| Gluconic lactone | 393 | 22.659267 | 2.953269316 |
| beta-Glutamic acid | 216 | 22.9179 | 0.152740063 |
| 4-hydroxycinnamic acid | 295 | 22.9386 | 0.126319823 |
| azelaic acid | 201 | 23.207533 | 0.794847225 |
| shikimic acid | 204 | 23.641667 | 105.2242812 |
| mucic acid | 333 | 23.6676 | 25.98921681 |
| citric acid | 211 | 23.778333 | 41.53266734 |
| isocitric acid | 245 | 23.863067 | 5.045483285 |
| fructose | 103 | 24.707067 | 21.15488681 |
| quinic acid | 240 | 24.727833 | 0.433773258 |
| D-Talose | 160 | 25.542467 | 100.616371 |
| myo-inositol | 204 | 25.5848 | 458.7421436 |
| galactose | 204 | 25.8728 | 15.40164393 |
| Analyte 289 | 203 | 25.9999 | 3.727588167 |
| adrenaline | 203 | 26.1893 | 24.27899964 |
| lysine | 174 | 26.279233 | 99.24161968 |
| mannitol | 205 | 26.447 | 98.2008711 |
| glucuronic acid | 160 | 26.549033 | 1.516076103 |
| sorbitol | 217 | 26.725467 | 3.073098495 |
| D-galacturonic acid | 160 | 26.789467 | 2.89078006 |
| N-Acetyl-beta-D-mannosamine | 159 | 26.7012 | 2.048191712 |
| iminodiacetic acid | 232 | 26.939933 | 1.918093531 |
| Allo-inositol | 191 | 27.0195 | 0.734239833 |
| N-formyl-L-methionine | 175 | 27.061 | 1.583529589 |
| Isopropyl-beta-D-thiogalactopyranoside | 217 | 27.0999 | 23.75197788 |
| 2-Butyne-1,4-diol | 240 | 27.40085 | 0.017042928 |
| Threitol | 103 | 27.5349 | 8.413842196 |
| pantothenic acid | 247 | 27.872133 | 0.242953598 |
| gluconic acid | 333 | 28.077967 | 28.22540841 |
| Saccharic acid | 295 | 28.335667 | 0.439653235 |
| beta-Mannosylglycerate | 204 | 28.896067 | 303.1107899 |
| palmitic acid | 117 | 29.233267 | 6.619730694 |
| Analyte 337 | 304 | 30.1067 | 1.081524236 |
| Analyte 338 | 72 | 30.1249 | 9.436885312 |
| allose | 205 | 30.9239 | 4.522603245 |
| phytosphingosine | 204 | 32.292867 | 5.834367758 |
| N-Acetyltryptophan | 202 | 32.343 | 45.71624563 |
| D-(glycerol 1-phosphate) | 299 | 32.5817 | 0.525303581 |
| tryptophan | 202 | 32.6466 | 73.62142297 |
| uridine | 261 | 32.7607 | 0.232717627 |
| trans-4-hydroxy-L-proline | 376 | 33.159367 | 2.686447488 |
| L-dopa | 218 | 33.3392 | 3.729493753 |
| stearic acid | 188 | 33.4586 | 0.956832254 |
| 2-Deoxy-D-galactose | 204 | 33.472433 | 4.914556635 |
| 2-deoxy-D-glucose | 355 | 33.809667 | 0.041759036 |
| Galactinol | 204 | 33.792367 | 613.7553225 |
| Glucosaminic acid | 295 | 34.063933 | 0.317563881 |
| 6-phosphogluconic acid | 225 | 34.84135 | 0.12752602 |
| Tagatose | 103 | 35.136233 | 1.659879076 |
| D-erythro-sphingosine | 233 | 35.57815 | 0.34989793 |
| d-Glucoheptose | 262 | 35.506367 | 3.947225192 |
| Digalacturonic acid | 204 | 35.9249 | 22.70918625 |
| noradrenaline | 174 | 36.8701 | 0.315628819 |
| xanthosine | 325 | 37.1607 | 7.65879534 |
| N-Methyl-L-glutamic acid | 260 | 37.6873 | 2.009066821 |
| Cellobiotol | 204 | 38.176767 | 116.1897171 |
| 1-Kestose | 217 | 38.382567 | 32.20245457 |
| maltitol | 217 | 39.22575 | 1.423153211 |
| D-Altrose | 213 | 39.85095 | 0.173204268 |
| adenosine | 230 | 40.077567 | 20.43353227 |
| maltotriose | 240 | 40.976933 | 0.013995854 |
| raffinose | 240 | 41.364333 | 0.017208713 |
| 3,4-dihydroxybenzoic acid | 193 | 41.4698 | 1.612789302 |
| trehalose | 191 | 41.725833 | 81.58987266 |
| Leucrose | 204 | 42.121 | 1.558276482 |
| guanosine | 295 | 42.125333 | 1.013707287 |
| Sophorose | 243 | 42.370967 | 0.539777103 |
| maltose | 204 | 42.670133 | 1.754455031 |
| Turanose | 204 | 42.671 | 0.624429976 |
| palatinitol | 225 | 42.72805 | 0.008676714 |
| Gentiobiose | 160 | 42.8889 | 8.934474139 |
| salicin | 243 | 42.903633 | 0.189663469 |
| Isomaltose | 204 | 43.550467 | 9.645215981 |
| sucrose | 243 | 44.074533 | 0.447309818 |
| Vanillylmandelic acid | 219 | 44.309767 | 0.89943947 |
| Melezitose | 169 | 45.152033 | 0.237917841 |
| Palatinose | 261 | 45.1382 | 0.011128769 |
| Chlorogenic Acid | 256 | 45.8248 | 0.108344041 |

**Supplementary Table 2. The interaction information of target protein and active components in *Lobelia chinensis***

| Uniport | Target protein | Full name of target protein | Active components |
| --- | --- | --- | --- |
| P06401 | PGR | Progesterone receptor | (3S,8S,9S,10R,13R,14S,17R)-17-[(1S,4R)-4-ethyl-1,5-dimethylhexyl]-10,13-dimethyl-2,3,4,7,8,9,11,12,14,15,16,17-dodecahydro-1H-cyclopenta[a]phenanthren-3-ol |
| P07550 | ADRB2 | Beta-2 adrenergic receptor | lobelanidine  Lobelanine  2-[(2R,6S)-6-[(2R)-2-hydroxy-2-phenylethyl]-1-methylpiperidin-2-yl]-1-phenylethanone  scoparone  Limetin  isoferulic acid  acacetin |
| P08238 | HSP90 | Heat shock protein HSP 90 | Hesperetin  (+)-medioresinol  acacetin |
| P09917 | ALOX5 | Arachidonate 5-lipoxygenase | Leptodactylone |
| P09960 | LTA4H | Leukotriene A-4 hydrolase | scoparone  Limetin  isoferulic acid |
| P13500 | CCL2 | C-C motif chemokine 2 | scoparone |
| P23219 | PTGS1 | Prostaglandin G/H synthase 1 | norlobelanine  Leptodactylone  scoparone  Hesperetin  Limetin  isoferulic acid  HMF  acacetin |
| P27487 | DPP4 | Dipeptidyl peptidase IV | Leptodactylone  HMF  acacetin |
| P29474 | NOS3 | Nitric-oxide synthase, endothelial | Leptodactylone  Limetin  isoferulic acid  (+)-medioresinol |
| P35354 | PTGS2 | Prostaglandin G/H synthase 2 | Leptodactylone  scoparone  Hesperetin  Limetin  isoferulic acid  HMF  (+)-medioresinol  acacetin |
| Q8TGA1 | FAS | Fatty acid synthase | acacetin |
| P78536 | ADAM17 | Disintegrin and metalloproteinase domain-containing protein 17 | Norlobelanine  Leptodactylone |
| P30542 | ADORA1 | Adenosine receptor A1 | Lobelanidine  4-oxoniobenzoate(PHB) |
| P29274 | ADORA2A | Adenosine receptor A2a | lobelanidine  2-[(2R,6S)-6-[(2R)-2-hydroxy-2-phenylethyl]-1-methylpiperidin-2-yl]-1-phenylethanone |
| P13945 | ADRB3 | Beta-3 adrenergic receptor | Leptodactylone  5-Hydroxymethylfurfural(HMF) |
| O60218 | AKR | Aldo-keto reductase | Leptodactylone  5-Hydroxymethylfurfural(HMF)  acacetin |
| P31749 | AKT1 | RAC-alpha serine/threonine-protein kinase | acacetin |
| P15144 | ANPEP | Aminopeptidase N | acacetin |
| Q16853 | AOC3 | Membrane primary amine oxidase | Lobelanidine  Lobelanine |
| Q43295 | APK1 | Adenylyl-sulfate kinase 1 | 2-[(2R,6S)-6-[(2R)-2-hydroxy-2-phenylethyl]-1-methylpiperidin-2-yl]-1-phenylethanone；  scoparone |
| P09871 | C1S | Complement C1s subcomponent | 4-oxoniobenzoate(PHB)；  Hesperetin |
| P19256 | CD58 | Lymphocyte function-associated antigen 3 | isoferulic acid;  (+)-medioresinol |
| P06731 | CEACAM5 | Carcinoembryonic antigen-related cell adhesion molecule 5 | acacetin |
| P23946 | CMA1 | Chymase | 4-oxoniobenzoate(PHB);  Leptodactylone;  isoferulic acid |
| Q92523 | CPT1B | Carnitine O-palmitoyltransferase 1 | Leptodactylone;  5-Hydroxymethylfurfural(HMF) |
| P53674 | CRYBB1 | Beta-crystallin B1 | 2-[(2R,6S)-6-[(2R)-2-hydroxy-2-phenylethyl]-1-methylpiperidin-2-yl]-1-phenylethanone |
| P17812 | CTPS | CTP synthase 1 | Acacetin |
| P22413 | ENPP1 | Ectonucleotide pyrophosphatase/phosphodiesterase family member 1 | Acacetin |
| Q9H6S3 | EPS8L2 | Epidermal growth factor receptor kinase substrate 8-like protein 2 | Acacetin |
| P11474 | ESRRA | Steroid hormone receptor ERR1 | Acacetin |
| P55085 | F2RL1 | Proteinase-activated receptor 2 | Lobelanidine;  Hesperetin;  5-Hydroxymethylfurfural(HMF) |
| O75844 | FACE | CAAX prenyl protease 1 homolog | Acacetin |
| P01275 | GCG | Glucagon | Leptodactylone;  5-Hydroxymethylfurfural(HMF) |
| Q04760 | GLO1 | Lactoylglutathione lyase | Acacetin |
| P43220 | GLP1R | Glucagon-like peptide 1 receptor | Acacetin;  5-Hydroxymethylfurfural(HMF) |
| P49841 | GSK3B | Glycogen synthase kinase-3 beta | Leptodactylone;  5-Hydroxymethylfurfural(HMF)  Acacetin |
| Q9NWT6 | HIF1AN | Hypoxia-inducible factor 1-alpha inhibitor | Acacetin |
| P17710 | HK1 | Hexokinase-1 | Leptodactylone;  5-Hydroxymethylfurfural(HMF)  Acacetin |
| P41235 | HNF4A | Hepatocyte nuclear factor 4-alpha | Leptodactylone;  5-Hydroxymethylfurfural(HMF)  Acacetin |
| Q9Y5N1 | HRH3 | Histamine H3 receptor | (+)-medioresinol;  isoferulic acid;  scoparone |
| P80365 | HSD11B2 | Corticosteroid 11-beta-dehydrogenase isozyme 2 | Leptodactylone;  5-Hydroxymethylfurfural(HMF) |
| P13597 | ICAM1 | Intercellular adhesion molecule 1 | Lobelanine;  norlobelanine |
| P08069 | IGF1R | Insulin-like growth factor 1 receptor | Acacetin |
| O14920 | IKBKB | Inhibitor of nuclear factor kappa-B kinase subunit beta | Lobelanine;  2-[(2R,6S)-6-[(2R)-2-hydroxy-2-phenylethyl]-1-methylpiperidin-2-yl]-1-phenylethanone |
| P06213 | INSR | Insulin receptor | Acacetin |
| IRAK4 | IRAK4 | Interleukin-1 receptor-associated kinase 4 | 4-oxoniobenzoate(PHB);  Limetin |
| P13612 | ITGA4 | Integrin alpha-4 | 5-Hydroxymethylfurfural(HMF)  Acacetin |
| P08648 | ITGA5 | Integrin alpha-5 | 5-Hydroxymethylfurfural(HMF)  Acacetin |
| P05556 | ITGB1 | Integrin beta-1 | Acacetin |
| P48048 | KCNJ1 | ATP-sensitive inward rectifier potassium channel 1 | Acacetin;  Leptodactylone;  5-Hydroxymethylfurfural(HMF) |
| Q14654 | KCNJ11 | ATP-sensitive inward rectifier potassium channel 11 | Acacetin;  Leptodactylone;  5-Hydroxymethylfurfural(HMF) |
| P10721 | KIT | Mast/stem cell growth factor receptor Kit | 2-[(2R,6S)-6-[(2R)-2-hydroxy-2-phenylethyl]-1-methylpiperidin-2-yl]-1-phenylethanone;  Limetin;  4-oxoniobenzoate(PHB) |
| Q9P0G3 | KLKL6 | Kallikrein-14 | Leptodactylone;  Limetin;  Acacetin |
| P19256 | LFA3 | Lymphocyte function-associated antigen 3 | Leptodactylone;  Scoparone;  Hesperetin;  Limetin |
| Q16539 | MAPK14 | Mitogen-activated protein kinase 14 | Lobelanidine;  Hesperetin;  5-Hydroxymethylfurfural(HMF) |
| P45984 | MAPK9 | Mitogen-activated protein kinase 9 | 5-Hydroxymethylfurfural(HMF)  Hesperetin;  Norlobelanine |
| O43451 | MGAM | Maltase-glucoamylase | acacetin |
| P14174 | MIF | Macrophage migration inhibitory factor | Acacetin;  Scoparone;  norlobelanine |
| P50280 | MMP7 | Matrilysin | Leptodactylone;  Scoparone;  Hesperetin |
| P22894 | MMP8 | Neutrophil collagenase | Leptodactylone;  Scoparone;  Hesperetin |
| P49146 | NPY2R | Neuropeptide Y receptor type 2 | Leptodactylone  5-Hydroxymethylfurfural(HMF)  acacetin |
| P11926 | ODC1 | Ornithine decarboxylase | Acacetin  2-[(2R,6S)-6-[(2R)-2-hydroxy-2-phenylethyl]-1-methylpiperidin-2-yl]-1-phenylethanone |
| P09874 | PARP1 | Poly [ADP-ribose] polymerase 1 | Acacetin |
| O15530 | PDPK1 | 3-phosphoinositide-dependent protein kinase 1 | Acacetin |
| P42336 | PIK3CA | Phosphatidylinositol 4,5-bisphosphate 3-kinase catalytic subunit alpha isoform | isoferulic acid  Acacetin  Hesperetin |
| P05771 | PKCB | Protein kinase C beta type | Acacetin |
| P04054 | PLA2A | Phospholipase A2 | 4-oxoniobenzoate(PHB)  Leptodactylone |
| P47712 | PLA2G4A | Cytosolic phospholipase A2 | Acacetin |
| Q13393 | PLD1 | Phospholipase D1 | 2-[(2R,6S)-6-[(2R)-2-hydroxy-2-phenylethyl]-1-methylpiperidin-2-yl]-1-phenylethanone  Acacetin |
| P00491 | PNP | Purine nucleoside phosphorylase | Acacetin |
| Q03181 | PPARD | Peroxisome proliferator-activated receptor delta | Scoparone  Hesperetin  Acacetin |
| P43088 | PTGFR | Prostaglandin F2-alpha receptor | Scoparone  Limetin |
| Q9P2B2 | PTGFRN | Prostaglandin F2 receptor negative regulator | Scoparone  Limetin |
| P43119 | PTGIR | Prostacyclin receptor | Acacetin |
| Q92729 | PTPRU | Receptor-type tyrosine-protein phosphatase U | Leptodactylone  Acacetin |
| P06737 | PYGL | Glycogen phosphorylase, liver form | Leptodactylone  Acacetin |
| P05121 | SERPINE1 | Plasminogen activator inhibitor 1 | Leptodactylone  Acacetin |
| P08842 | STS | Steryl-sulfatase | Acacetin |
| P43405 | SYK | Tyrosine-protein kinase SYK | isoferulic acid  Acacetin |
| Q02763 | TEK | Angiopoietin-1 receptor | Acacetin |
| P01375 | TNF | Tumor necrosis factor | Norlobelanine  isoferulic acid  Acacetin |
| P19438 | TNFRSF1A | Tumor necrosis factor receptor superfamily member 1A | 5-Hydroxymethylfurfural(HMF)  Acacetin |
| P17948 | VGFR1 | Vascular endothelial growth factor receptor 1 | Norlobelanine  Acacetin |


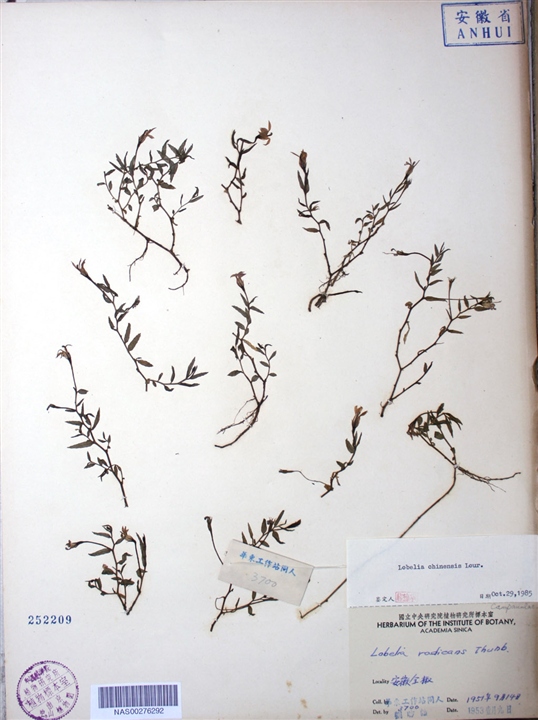


**Supplementary Figure S1. The specimen information of *Lobelia chinensis***

**Supplementary Figure S2. The typical total ion current (TIC) of *Lobelia chinensis***


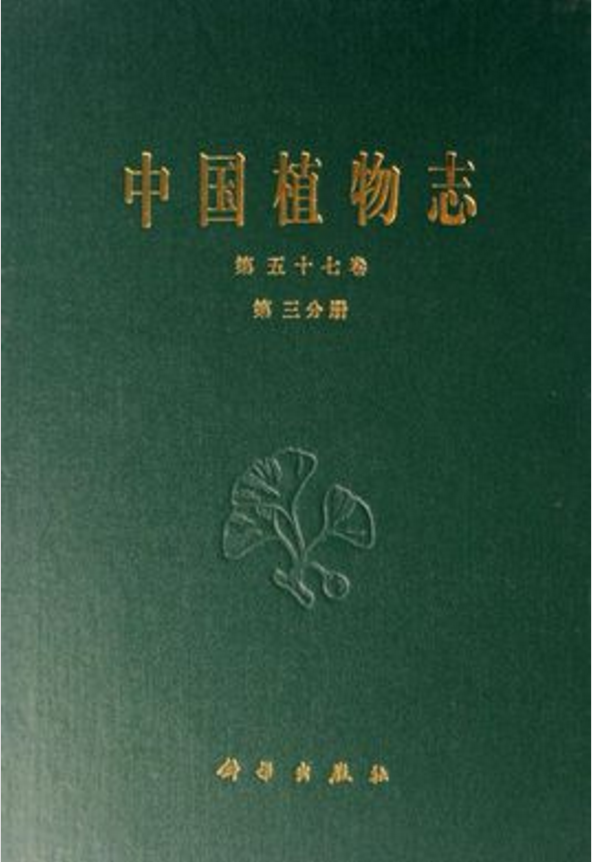

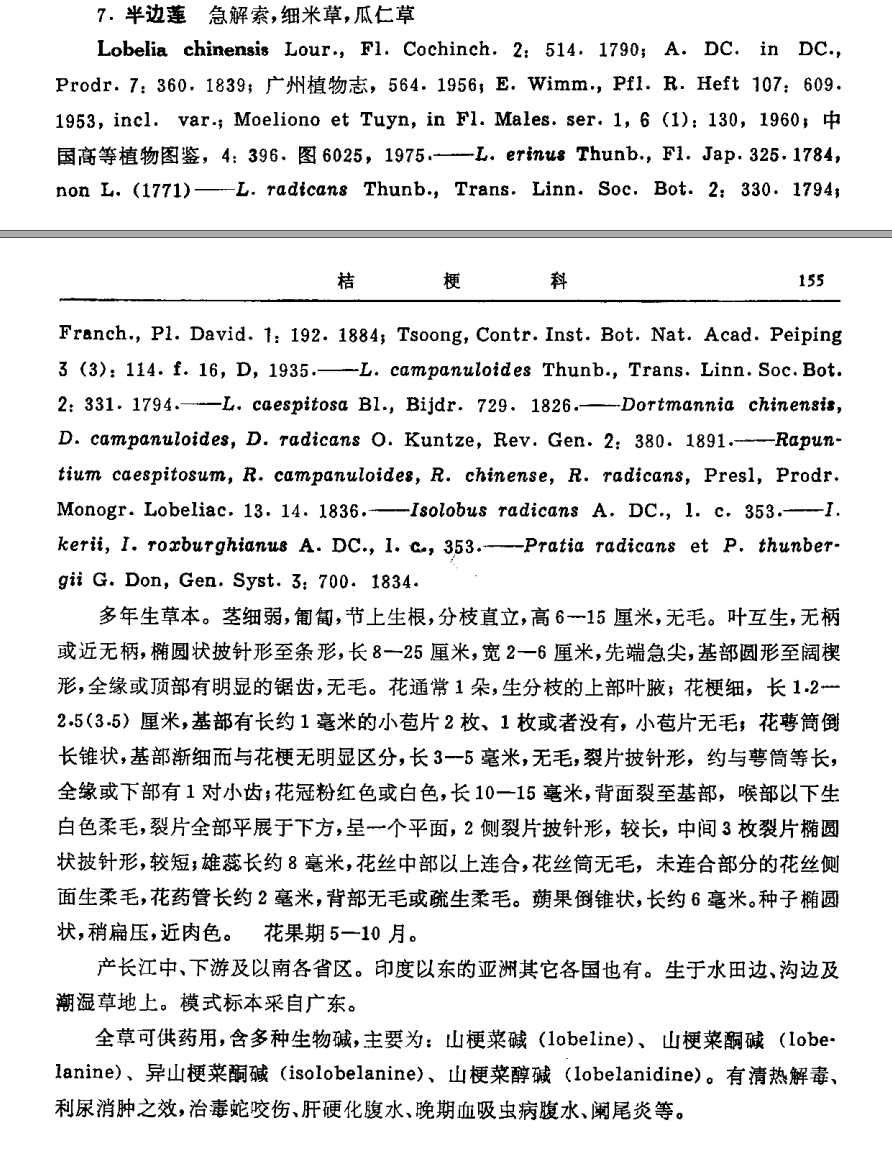


**Supplementary Figure S3. Flora of China**
